# Home and Out-of-Hospital Therapy with COVID-19 Convalescent Plasma in Europe

**DOI:** 10.3390/life12111704

**Published:** 2022-10-26

**Authors:** Daniele Focosi, Massimo Franchini

**Affiliations:** 1 North-Western Tuscany Blood Bank, Pisa University Hospital, 56124 Pisa, Italy; 2 Division of Hematology and Transfusion Medicine, Carlo Poma Hospital, 46100 Mantua, Italy

**Keywords:** COVID-19 convalescent plasma, outpatients, legislation, neutralizing antibodies

## Abstract

COVID19 convalescent plasma (CCP) has proven an effective treatment for outpatients, and CCP collected from vaccinated donors is among the few effective therapeutic options for immunocompromised patients. Despite this, most countries are still relying over in-hospital compassionate usages outside clinical trials. Given the need for early treatment, home transfusions are expecially needed. We review here the state of the art for out-of-hospital CCP transfusions and discuss solutions to potential burocratic hurdles.

In December 2019, the Severe Acute Respiratory Syndrome Coronavirus 2 (SARS-CoV-2) initiated the pandemic of the coronavirus disease-19 (COVID-19), accounting for nearly 620 million infected people worldwide and more than 6.5 million deaths as of 22 September 2022 [1]. Italy has been the first western country to be hit by this respiratory viral infection, which led to an unprecedented severe health and social crisis with more than 22 million cases and nearly 180,000 deaths at the time of writing. At the beginning of the pandemic, several therapies were tried as antiviral drugs against SARS-CoV-2, including chloroquine, lopinavir/ritonavir, and ribavirin, but there was no success. As convalescent plasma had been used in other infectious disease outbreaks (ranging from Spanish influenza A in 1918 to SARS epidemic in 2002–2003, and the Ebola epidemic in 2016 [2]), collection and transfusion of COVID-19 convalescent plasma (CCP) was rapidly deployed worldwide to treat patients with COVID-19 [3]. Various mechanisms have been suggested as responsible for the therapeutic effect of CCP, such as anti-inflammatory and immunomodulatory properties. However, most of the antiviral effects of CCP are related to neutralizing antibodies to SARS-CoV-2, able to block viral replication and disease progression [4]. Despite numerous studies assessing the clinical use of CCP, their results have been quite controversial, overall reflecting great inter-study methodological heterogeneities and inconsistencies, along with a poorly standardized biological product, such as CCP [5]. Despite these limitations, a consistent amount of literature supported the clinical benefit of this antibody-based treatment when administered early (within 72 h since onset of symptoms) and with high titer of neutralizing antibodies [6]. An overview of systematic reviews (umbrella review) on CCP efficacy in COVID-19, including 29 systematic reviews based on 53 unique primary studies (17 randomized controlled trials (RCTs) and 36 non-RCT), found a mortality reduction in CCP over standard therapy when administered early and at high titer, without increased adverse reactions [7]. In a recent systematic review including 30 RCTs on CCP use, we analyzed variables associated with efficacy, such as clinical settings, disease severity, CCP SARS-CoV-2 nAb levels and function, dose, timing of administration (variously defined as time from onset of symptoms, molecular diagnosis, diagnosis of pneumonia, or hospitalization, or by serostatus), outcomes (defined as hospitalization, requirement for ventilation, clinical improvement, or mortality), CCP provenance and time since collection, and criteria for efficacy [8]. After a careful literature screening, we observed that when the analysis of the studies was restricted to those subgroups of patients treated early and with high titer CCP, most of the inter-studies discrepancies disappeared, the great majority of them showing signals of efficacy after a proper CCP use.

Another important issue regards the CCP safety. Fresh frozen plasma (FFP) is a well-known blood component which has been transfused to millions of patients, at every age and in many clinical conditions, worldwide, for more than 60 years. A recent pilot RCT on the use of FFP in hospital setting found that secondary outcomes including adverse events were uncommon [9]. Although this solid clinical experience had unequivocally documented the low rate of severe adverse reactions, and the absolute safety of hyperimmune plasma had been assessed during the past epidemics, no safety data on CCP were available at the beginning of the SARS-CoV-2 pandemic. To address this issue, we have performed a systematic literature review that includes 30 studies (14 RCT and 16 matched control studies) [10]. The mean prevalence of all and severe CCP-infusion related adverse events was 2.1% and 0.7%, respectively. In addition, treatment with CP did not increase the risk of overall adverse events (risk difference, RD: 0.01; 95% CI, −0.02/0.03; *p* = 0.65) and severe adverse events (RD: 0.00; 95% CI, −0.03/0.03; *p* = 0.81) compared with standard treatment. Similarly, the rate of thromboembolic events did not differ between the two study groups (1.4% in the CP arm versus 1.7% in the control arm; RD 0.00; 95% CI, −0.01/0.03; *p* = 0.24). This data definitively documented the safety of CCP as treatment of COVID-19 patients.

During the first pandemic wave (February 2020–July 2020), the US Food and Drug Administration (FDA) authorized CCP for emergency use because of its safety, and this permitted the transfusion with CCP of more than 500,000 COVID-19 patients [11]. In Europe, although the European Commission promoted the SUPPORT-e (SUPPORTing high quality evaluation of COVID-19 convalescent plasma throughout Europe) project, clinical use of CCP has always been considered experimental and thus restricted to hospitalized patients in the frame of experimental studies under local ethical committee authorization. This requirement greatly hampered a wider CCP use in European countries, including Italy. In Italy, indeed, the authorization for use of CCP remains difficult to obtain, requiring multiple permissions (local ethical committees, hospital health management, etc.), and often discouraging the bravest of physicians. However CCP use, which gradually decreased since the beginning of 2021 following the marketing of the more potent monoclonal antibodies (mAbs) against COVID-19, has recently renewed due to the advent of the last SARS-CoV2 variant, named B.1.1.529 (Omicron), which escapes most, if not all, mAbs authorized for therapy [12]. The Omicron variant is especially dangerous for immunocompromised patients, who are not able to mount a sufficiently protective antibody response to vaccine or prior infection, and are therefore left at higher risk of morbidity and mortality for COVID-19 [13]. A recent systematic review and meta-analysis, including 5 RCTs and 4 matched controlled studies conducted in immunocompromised COVID-19 patients, has shown a clinical benefit (risk ratio 0.65) from CCP versus standard care [14]. For such reasons, several national and international scientific societies currently include CCP among the possible therapies for COVID-19 in patients with hematological or solid cancers or other underlying congenital or acquired causes of immunosuppression.

Apart from the restricted use in immunocompromised patients, there is a new line of evidence from recent RCTs showing that the CCP transfusion in COVID-19 patients before hospitalization has a great beneficial effect on improving the disease’ course, dramatically reducing the need for hospital admission, and finally reducing COVID-19-related patients’ mortality. This finding is not surprising given that CCP, as any other antiviral (including mAbs), provides its best efficacy when given early in disease course [15]. Overall, 5 large RCTs have been conducted on CCP as outpatient therapy for COVID-19. The first published RCT was from Argentina where 160 older adult patients with mild COVID-19 were randomized to receive CCP or placebo [16] early (within 72 h after the onset of symptoms). In the intention-to-treat analysis, the risk for respiratory disease was almost halved in patients receiving CCP compared with those in the placebo arm (relative risk, RR: 0.40; 95% CO, 0.20–0.81). The administration of CCP was not associated with any severe adverse events. This study was followed by another RCT from the USA (C3PO trial) which enrolled 514 patients (254 in CCP arm and 254 in placebo arm) [17]. Although adverse events occurred with similar frequency in both groups, no CCP-related beneficial effects of early administration (within one week from COVID-19 symptom onset) of CCP on disease progression (30% in CCP group versus 31.9% in placebo group) were, however, observed in the entire cohort, but reappeared when patients hospitalized the same day of recruitment were excluded from analysis [18]. A similar conclusion was made by another RCT from Spain (CONV-ert trial) which recruited 376 patients randomly assigned to high titer methylene blue-treated CCP (*n* = 188) or placebo (*n* = 188) [19]. No differences in the hospitalization rate were found in CCP versus placebo arms (12% vs. 11%) with concerns about damage of Fc-mediated functions by methylene blue [20]. A fourth RCT was run in the Netherlands (COV-Early), published first as a combined analysis with the Spanish RCT [21], and later as single study [22]. Among the 797 COVID-19 outpatients included, 390 received CP and 392 received the placebo. CCP effect on hospital admission or death was the largest in patients with ≤5 days from symptoms (odds ratio, OR 0.658, 95% CI 0.394–1.085). The fifth RCT was conducted in the USA by Sullivan and colleagues [23] on 1181 outpatients with recent-onset COVID-19. The primary outcome (i.e., hospitalization) occurred in 17 of 592 participants (2.9%) who received CCP and in 37 of 589 participants (6.3%) who received control plasma (absolute risk reduction, 3.4 percentage points; 95% confidence interval, 1.0 to 5.8; *p* = 0.005), which corresponded to a relative risk reduction of 54%. Again, the rate of severe (grade 3 and 4) adverse events did not differ in the two groups (7 in CCP-treated group and 9 in control plasma group). All these data were summarized in a recent systematic review pre-published by Sullivan and colleagues [15], which compared the efficacy all antiviral regimens of different classes to reduce COVID-19 hospitalizations. The authors concluded that, considering the dramatically emerging resistance of Omicron sublineages to currently commercialized mAbs, the early use of CCP from vaccinated donors with high titer nAb titers should be re-evaluated to reduce the risk of hospitalization in COVID-19 patients at risk of disease progression.

Early CCP delivery implies out-of-hospital administration, which often translates into home therapy (HT). Regarding the safety of CCP administration in outpatient setting, there are no particular reasons of concern considering the large amount of data documenting the safety of both FFP and CCP in hospitalized patients. In a survey conducted by the International Society of Blood Transfusion (ISBT) about home and ambulatory use of CCP, Al-Riyami et al. showed that HT was practiced in Australia, the UK, Belgium, France, Japan, Nigeria, the Netherlands, Spain, Italy, Norway, the United States, and some provinces in Canada [24]. We note that in both the abstract and the main text, Italy is reported to have implemented CCP home therapy [24]. In Supplementary Table S1, Italy is not listed among the 19 respondents, and accordingly it does not appear with results in Supplementary Table S2 [24]. Hence, we wonder which was the response of the Italian National Blood Center, acknowledged by the authors as a contributor. As practicing transfusion physicians in Italy, we are not aware of any CCP home or ambulatory treatment in Italy, and we would like to know the sites and number of treated patients reported in that survey, if any.

Regardless of the Italian case, we feel that the root cause for the lack of home or outpatient CCP use in Europe stems from the current European Directorate for Quality of Medicines and Healthcare (EDQM) guidelines [25]. At the beginning of the pandemic, we noted that current EDQM regulations do not recognize convalescent plasma as a separate blood component, and the related legislations of EU member countries treat it as regular plasma: this means it has to fulfil the same requirements, largely aimed at preserving labile clotting factors (freezing time and temperatures, storage, expiry after thawing) [26], while instead, neutralizing antibodies, the main active ingredient of CCP, are much more stable, even at room temperature [27]. Accordingly, member countries can only prescribe CCP as an experimental blood component for compassionate usage, i.e., after case-by-case positive opinion by the local ethical committee. This status has not changed in the last 3 years neither in Italy nor in member countries.

For example, in Italy, after the closure of RCTs, a prescriber should have filled tens of pages of forms for each patient, and be the only one allowed to administer the treatment. While this can be somewhat feasible in-hospital, most general practitioners (GP) and emergency room physicians (i.e., the frontline settings where COVID-19 patients are first visited), do not have the time nor the experience to cope with this: in the case of home transfusions, the GP should also have taken full legal and economical responsibility for the ensuing transfusion step. No dedicated hospital ambulatories have been ever created for outpatient CCP transfusions in Italy, as confirmed in recent press releases [28], which contrasts with what has been done for anti-Spike monoclonal antibodies of similar or inferior efficacy [29].

As such, we encourage the ISBT to start talks with EDQM to urgently include convalescent plasma among the authorized blood components, or to issue emergency use authorizations that avoid submission of duplicate files for individual patients. Without such simplification, convalescent plasma will remain scarcely deployed both in the current pandemic (when it deserves essential value for immunocompromised patients) and in future pandemics.

## Data Availability

Not applicable.

## References

[1] World Health Organization (WHO) Coronavirus Disease (COVID-19). https://www.who.int/emergencies/diseases/novel-coronavirus-2019.

[2] Mair-Jenkins J., Saavedra-Campos M., Baillie J.K., Cleary P., Khaw F.M., Lim W.S., Makki S., Rooney K.D., Nguyen-Van-Tam J.S., Beck C.R. (2015). The effectiveness of convalescent plasma and hyperimmune immunoglobulin for the treatment of severe acute respiratory infections of viral etiology: A systematic review and exploratory meta-analysis. J. Infect. Dis..

[3] Casadevall A., Grossman B.J., Henderson J.P., Joyner M.J., Shoham S., Pirofski L.A., Paneth N. (2020). The Assessment of Convalescent Plasma Efficacy against COVID-19. Med.

[4] Franchini M., Glingani C., Liumbruno G.M. (2021). Potential mechanisms of action of convalescent plasma in COVID-19. Diagnosis.

[5] Franchini M., Liumbruno G.M., Piacentini G., Glingani C., Zaffanello M. (2021). The Three Pillars of COVID-19 Convalescent Plasma Therapy. Life.

[6] Focosi D., Franchini M. (2021). COVID-19 convalescent plasma therapy: Hit fast, hit hard!. Vox Sang..

[7] Franchini M., Focosi D., Corsini F., Cruciani M. (2021). Safety and efficacy of convalescent plasma in COVID-19: An overview of systematic reviews. Diagnostics.

[8] Focosi D., Franchini M., Pirofski L.A., Burnouf T., Paneth N., Joyner M.J., Casadevall A. (2022). COVID-19 Convalescent Plasma and Clinical Trials: Understanding Conflicting Outcomes. Clin. Microbiol. Rev..

[9] Carson J.L., Ness P.M., Pagano M.B., Philipp C.S., Bracey A.W., Brooks M.M., Nosher J.L., Hogshire L., Noveck H., Triulzi D.J. (2021). Plasma trial: Pilot randomized clinical trial to determine safety and efficacy of plasma transfusions. Transfusion.

[10] Franchini M., Cruciani M. (2021). How Safe Is COVID-19 Convalescent Plasma?. Mayo Clin. Proc..

[11] Senefeld J.W., Johnson P.W., Kunze K.L., van Helmond N., Klassen S.A., Wiggins C.C., Bruno K.A., Golafshar M.A., Petersen M.M., Buras M.R. (2021). Access to and safety of COVID-19 convalescent plasma in the United States Expanded Access Program: A national registry study. PLoS Med..

[12] Focosi D., McConnell S., Casadevall A., Cappello E., Valdiserra G., Tuccori M. (2022). Monoclonal antibody therapies against SARS-CoV-2. Lancet Infect. Dis..

[13] Focosi D., Franchini M. (2021). Potential use of convalescent plasma for SARS-CoV-2 prophylaxis and treatment in immunocompromised and vulnerable populations. Expert Rev. Vaccines.

[14] Senefeld J.W., Franchini M., Mengoli C., Cruciani M., Zani M., Gorman E.K., Focosi D., Casadevall A., Joyner M.J. (2022). COVID-19 convalescent plasma for the treatment of immunocompromised patients: A systematic review. medRxiv.

[15] Sullivan D.J., Focosi D., Hanley D., Franchini M., Ou J., Casadevall A., Paneth N. (2022). Effective antiviral regimens to reduce COVID-19 hospitalizations: A systematic comparison of randomized controlled trials. medRxiv.

[16] Libster R., Pérez Marc G., Wappner D., Coviello S., Bianchi A., Braem V., Esteban I., Caballero M.T., Wood C., Berrueta M. (2021). Early High-Titer Plasma Therapy to Prevent Severe Covid-19 in Older Adults. N. Engl. J. Med..

[17] Korley F.K., Durkalski-Mauldin V., Yeatts S.D., Schulman K., Davenport R.D., Dumont L.J., El Kassar N., Foster L.D., Hah J.M., Jaiswal S. (2021). Early Convalescent Plasma for High-Risk Outpatients with Covid-19. N. Engl. J. Med..

[18] Longer Critique of SIREN-C3PO (Korley et al) from the CCPP19 Leadership Group. https://ccpp19.org/news/corrected%20C3PO.pdf.

[19] Alemany A., Millat-Martinez P., Corbacho-Monné M., Malchair P., Ouchi D., Ruiz-Comellas A., Ramírez-Morros A., Codina J.R., Simon R.A., Videla S. (2021). High-titre methylene-blue treated Convalescent Plasma as an early treatment for COVID-19 outpatients: A randomized clinical trial. Lancet Respir. Med..

[20] Focosi D., Casadevall A. (2022). Convalescent plasma in outpatients with COVID-19. Lancet Respir. Med..

[21] Millat-Martinez P., Gharbharan A., Alemany A., Rokx C., Geurtsvankessel C., Papageourgiou G., van Geloven N., Jordans C., Groeneveld G., Swaneveld F. (2022). Prospective individual patient data meta-analysis of two randomized trials on convalescent plasma for COVID-19 outpatients. Nat. Commun..

[22] Gharbharan A., Jordans C., Zwaginga L., Papageorgiou G., van Geloven N., van Wijngaarden P., den Hollander J., Karim F., van Leeuwen-Segarceanu E., Soetekouw R. (2022). Outpatient convalescent plasma therapy for high-risk patients with early COVID-19. A randomized placebo-controlled trial. Clin. Microbiol. Infect..

[23] Sullivan D., Gebo K., Shoham S., Bloch E., Lau B., Shenoy A., Mosnaim G., Gniadek T., Fukuta Y., Patel B. (2021). Early Outpatient Treatment for Covid-19 with Convalescent Plasma. N. Engl. J. Med..

[24] Al-Riyami A.Z., Estcourt L., Rahimi-Levene N., Bloch E.M., Goel R., Tiberghien P., Thibert J.B., Bruun M.T., Devine D.V., Gammon R.R. (2022). Early and out-of-hospital use of COVID-19 convalescent plasma: An international assessment of utilization and feasibility. Vox Sang..

[25] Council of Europe European Directorate for the Quality of Medicines & HealthCare. 20th Edition of the Guide to the Preparation, Use and Quality Assurance of Blood Components..

[26] Focosi D., Farrugia A. (2020). Urgent need to regulate convalescent plasma differently from thawed plasma. Transf. Med. Hemother..

[27] Focosi D., Moscato G., Pistello M., Maggi F. (2021). Kinetics of anti-SARS-COV2 spike protein IgG and IgA antibodies at 4 °C: Implications for convalescent plasma stability. Transfus. Med..

[28] Politi A., Fubini M. (2022). Plasma convalescente. La terapia efficace per gli immunodepressi che in Italia non decolla. FQMillennium.

[29] Focosi D., Tuccori M. (2022). Prescription of Anti-Spike Monoclonal Antibodies in COVID-19 Patients with Resistant SARS-CoV-2 Variants in Italy. Pathogens.

